# Systemic Therapy for Microsatellite Instability Small Bowel Adenocarcinoma With Mesenteric Vascular Embolism as Initial Symptom: A Case Report

**DOI:** 10.3389/fmed.2021.764233

**Published:** 2021-11-08

**Authors:** Zhongyi Dong, Xiang Xia, Zizhen Zhang

**Affiliations:** Department of Gastrointestinal Surgery, Renji Hospital, School of Medicine, Shanghai Jiao Tong University, Shanghai, China

**Keywords:** case reports, intestinal cancers, immune checkpoint inhibitor, mesenteric vascular occlusion, palliative care

## Abstract

**Background:** Small bowel adenocarcinoma are relatively rare tumors of the digestive system. Due to the lack of specific screening methods, patients are often diagnosed at an advanced stage. At present, there is no specific surgical guidance and chemotherapy regimen for small bowel adenocarcinoma. Here, we report a rare small bowel adenocarcinoma case with mesenteric vascular embolization and microsatellite instability, in which palliative surgery combined with chemotherapy and anti-Programmed cell death protein 1(PD-1) therapy resulted in complete remission.

**Case Presentation:** The patient was a 55-year-old man who was admitted for suspected small bowel adenocarcinoma combined with incomplete ileus, mesenteric vascular occlusion and distant metastasis. We performed palliative surgery to remove adenocarcinoma as well as relieve obstruction. Then according to the pathological and immunohistochemical results (Stage IV and microsatellite instability), we used XELOX regimen combined with anti-PD-1 therapy. In last 2 years follow up, this patient achieved complete remission.

**Conclusions:** The possibility of small intestinal tumor should be considered in patients with mesenteric vascular obstruction. PD-1 blockade is an effective therapy for small bowel adenocarcinoma with microsatellite instability.

## Introduction

Although the small intestine occupies most of the length and surface area of the digestive tract, primary small intestine cancer is still a relatively rare tumor of the digestive system, which accounts for only about 3% of cancers occuring in this system (1). The most affected segment was duodenum, including 46–82% of the cases, followed by jejunum (11–31%) and ileum (7–21%) (2). But unlike other cancers of the digestive system, such as stomach and esophagus, the incidence of small intestinal malignancies has continued to rise over the past decade (3). Small bowel adenocarcinoma is estimated to be accounting for about 30–45% of the total cases, with a 5-year overall survival rate in patients at stage IV of 42% (4). The advanced patients usually have a delayed clinical presentation and non-specific symptoms such as abdominal pain, nausea and gastrointestinal bleeding (2). Herein, we report a case of microsatellite-unstable (MSI-H) advanced small bowel adenocarcinoma with mesenteric vascular occlusion as the initial symptom which is quite unusual.

## Case Presentation

**Figure 1:**
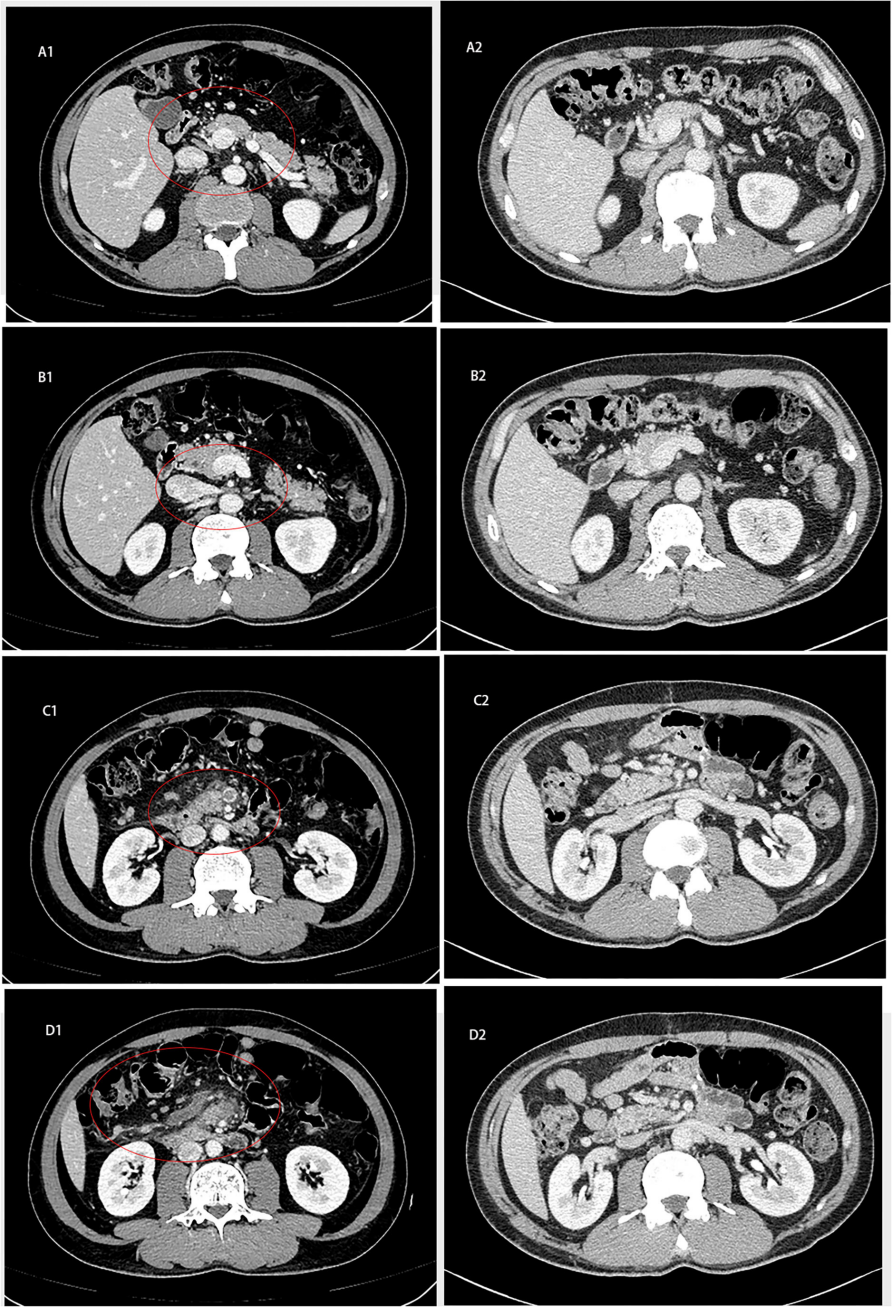
Abdominal computed tomography of patient. **(A1–D1)** Contrast-enhanced CT on February 2, 2020 showed that the small intestine in the right middle abdomen was occupied by tumor with multiple enlarged lymph nodes. **(A2–D2)** Abdominal CT on October 6, 2020 showed that the obstruction of intestinal lumen was relieved after operation 2020.

Without apparent inducement, a 55-year-old male patient was admitted for upper-middle abdominal pain with acid reflux, fullness and weakness for 1 week. Contrast-enhanced Computed tomography (CT) of the abdomen showed a small bowel mass with multiple enlarged lymph nodes in the right middle abdomen. The superior and inferior mesenteric vein trunks and their major tributaries were nearly occluded at the level adjacent to the tumor [Figures 1(A1–D1)].

The patient had no previous noteworthy medical history. And the patient did not smoke or consume alcohol. There was no noteworthy family medical history, such as cancer or small intestinal diseases. The patient had been in good physical condition, and had a good spirit.

Physical examination revealed normal abdominal findings. The abdomen was soft and flat, with no gurgling, bloating or tenderness. All other vital signs were stable. Laboratory tests showed a decrease in neutrophil percentage (43.5%, Normal range: 50–70%), a decrease in hemoglobin (95 g/L, Normal range: 120–160 g/L), and an increase in platelet count (386 ^*^ 10^9^L, Normal range: 100–300^*^10^9^/L). Prothrombin time increased (14.30 s, Normal range: 11–13 s) and fibrinogen content increased (4.64 g/L, Normal range: 2–4 g/L). The special tumor markers of digestive system (Alpha-fetoprotein, Carbohydrate antigen199, Carcinoembryonic antigen, Carbohydrate antigen724) were in the normal range. In addition, on chest scan, scattered fibrous tissues and emphysema were found in both lungs.

**Figure 2:**
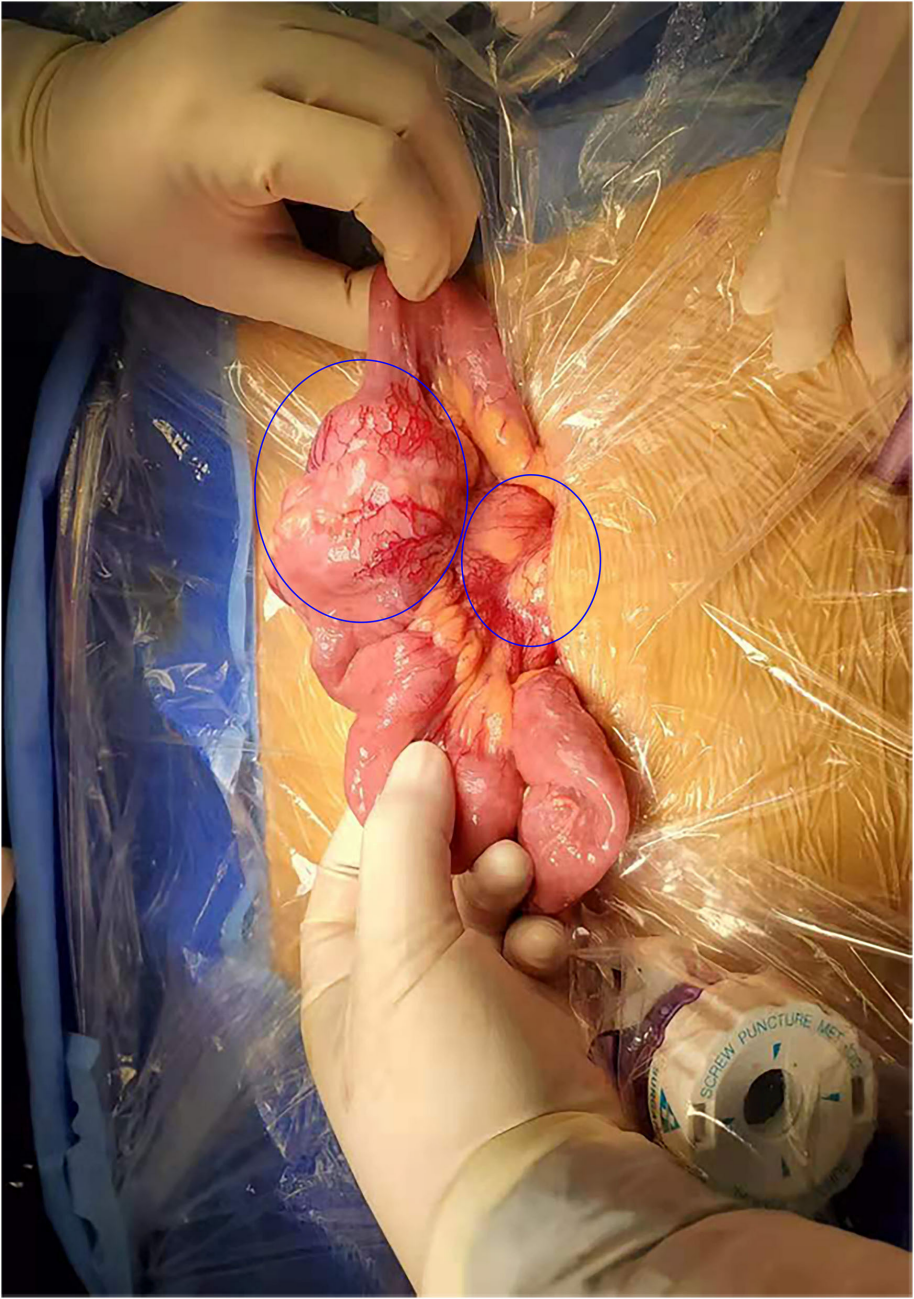
Surgical findings. Enlarged lymph nodes were identified in the root of the mesentery in addition to the mass tumor in the small intestine.

**Figure 3:**
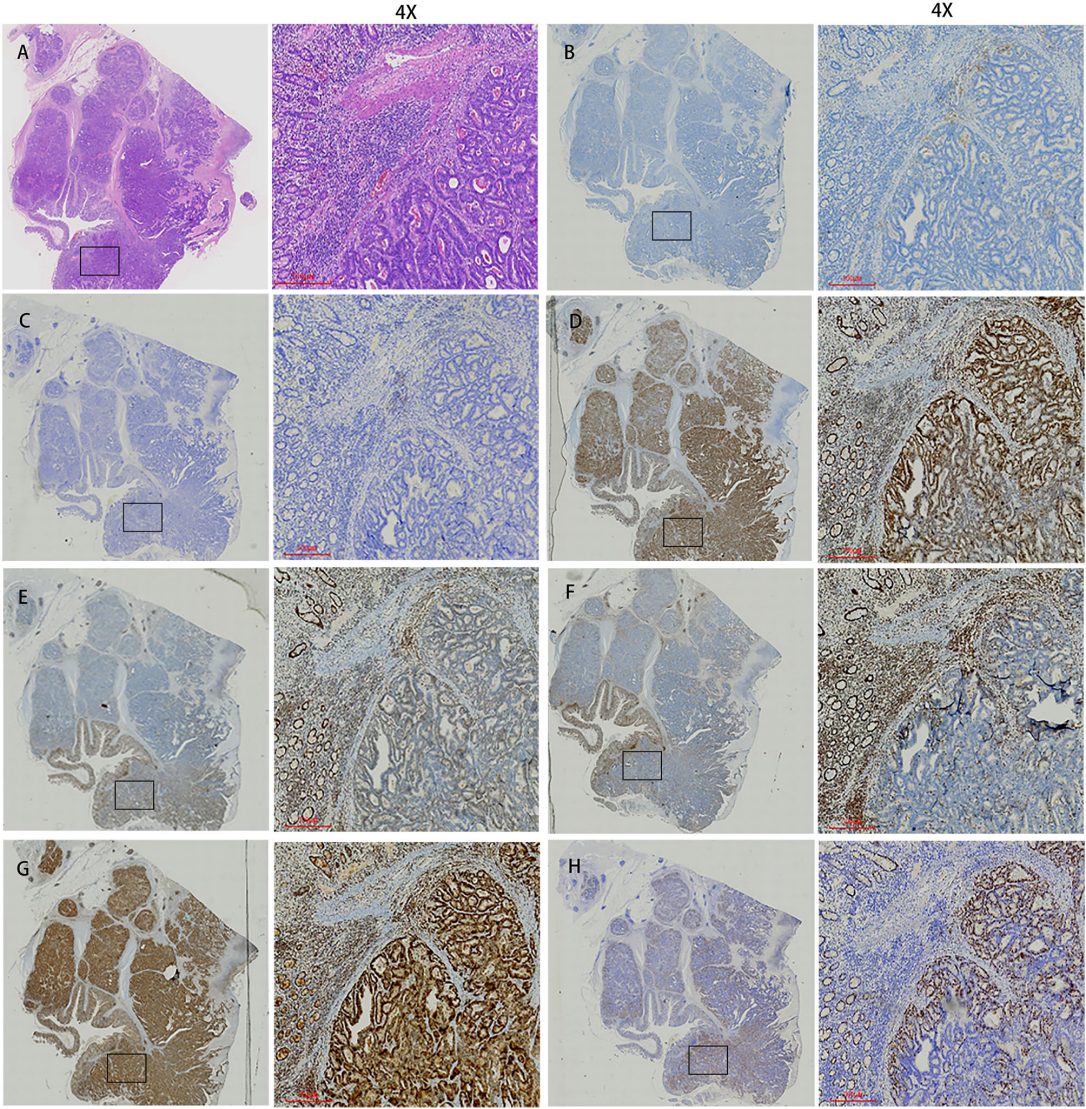
Histopathological findings and immunohistochemistry. **(A)** Histological findings showed tumor cells with cytoplasm rich in eosinophilic granules and clear nucleoli that showed dense proliferation. **(B)** PD-1. **(C)** PD-L1. **(D)** MLH1. **(E)** MSH2. **(F)** MSH6. **(G)** PMS2. **(H)** Ki67. The first image in each group was viewed from a macro perspective. The scale bars of second picture in each group was 300 μm.

The patient underwent a laparoscopic exploration and small bowel adenocarcinoma resection. The tumor was located in the small intestine 20 cm away from the ligament of Treitz. The size of the tumor was 6 ^*^ 5 ^*^ 5 cm. The tumor had invaded the serosa and caused incomplete obstruction. Meanwhile, multiple swollen lymph nodes could be found in the mesentery root and around the abdominal aorta, some of which has fused into a mass (Figure 2). Pathological specimens showed that this patient was grade II adenocarcinoma of the small intestine (7 ^*^ 4 ^*^ 1.3 cm), with the invasion of the serosa. Lymph nodes in the intestinal wall (1/1) showed metastasis and there were even three cancerous nodules. The upper and lower margins of the specimen were negative. Immunohistochemical results showed that cytokeratin CK7(–), cytokeratin CK20(+), Ki-67(70%), p53(+), MLH1(+), PMS2(+), MSH2(—/+), MSH6(–) (Figure 3).

By histopathology and imaging, the patient was considered to have metastatic adenocarcinoma of the small intestine. Immunohistochemical results showed that the MMR gene-associated protein was missing, indicating that it was microsatellite-unstable (MSI-H) small bowel adenocarcinoma and the anti-PD-1 therapy was presumed to be suitable. Based on the follow-up a month later, we treated with XELOX (Oxaliplatin 200 mg, St, IV; Capecitabine 1000 mg, BID, d1-14), taking a course of 3 weeks. The platinum drug was discontinued due to the toxicity after an eight-course of treatment, and Capecitabine continued to be administered orally alone for 10 courses. In addition, we combined the PD-1 inhibitor (200 mg, ST, IV) in 18 sessions. The patient was followed up regularly for 11 months after surgical resection, and there was no evidence of recurrence. After intensive care by medical staff and family, the patient's condition was greatly relieved. Now he was considered to be in complete remission according to the Fluorodeoxyglucose-positron emission tomography (FDG-PET) image and other assessment [Figures 1(A2–D2), 4].

**Figure 4:**
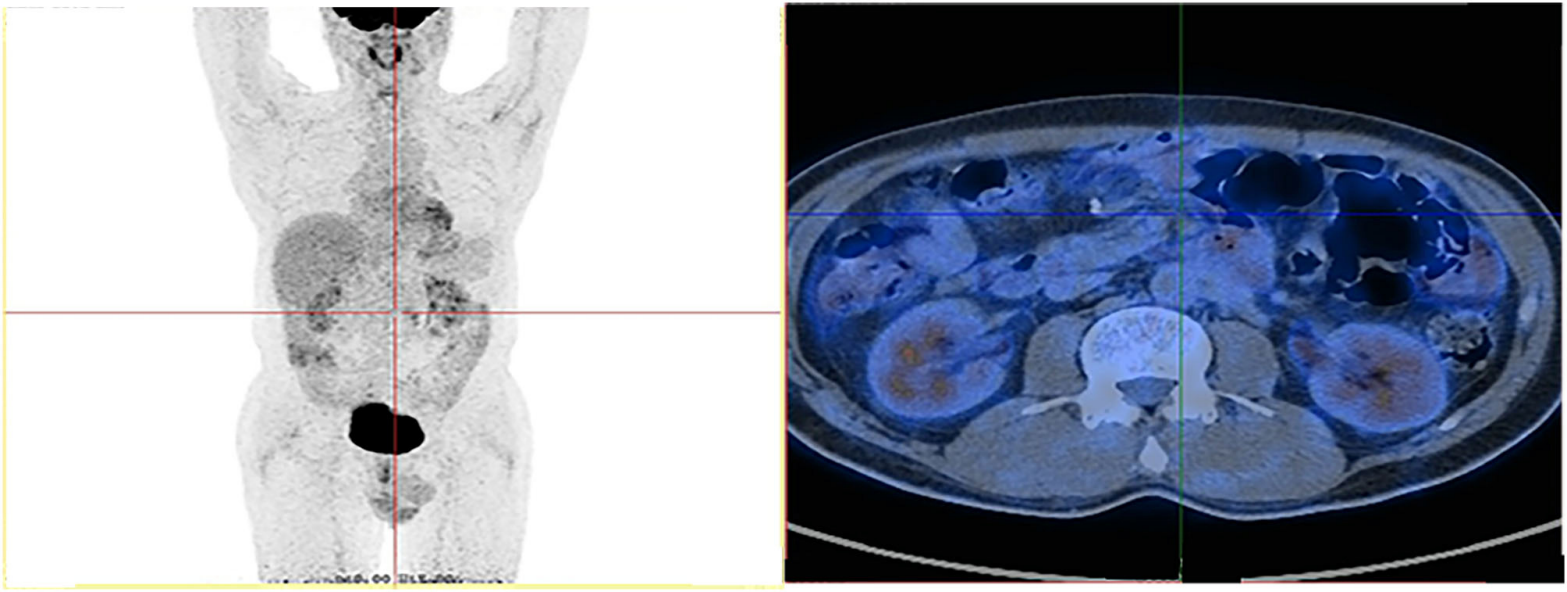
FDG-PET scan shows no metastatic lesions and lymph node.

## Discussion and Conclusion

Small intestinal malignancies are still rare, but due to the improvement of medical technology, the detection rate of these malignancies has been increasing in recent years (5). There is a high degree of diversity in the anatomic distribution and histological morphology of small intestinal malignant tumors. Small bowel adenocarcinoma is one of the most common types and it is usually found at the advanced stage due to the absence of obvious symptoms and the lack of specific testing methods (6). Lymph node metastasis and hematogenous metastasis are the most common forms of small bowel adenocarcinoma. But the small intestinal mucosa is rich in lymph node tissue, so it is prone to metastasize through lymph nodes in the early stage. The five-year survival rate was higher when the tumor was localized (85%), but the rate was only 3–5% for patients with distant metastasis (stage IV) (7).

Due to the low incidence of small bowel adenocarcinoma and the limited data available, its pathogenesis is still unknown but the risk factors are considered to be similar to those of colorectal cancer. Poor lifestyle, eating habits and some special diseases such as smoking, alcohol abuse, higher BMI, inflammatory bowel disease, inherited cancer syndrome, all increase the risk (2). The researchers compared changes in DNA copy number between small bowel cancer, stomach cancer and colorectal cancer, and found that small bowel adenocarcinoma was more similar to colorectal cancer (8). The above reasons lead to the fact that most of the existing treatment for the small bowel malignancies refer to colorectal cancer.

This case, a 55-year-old man, had suspected multiple metastases in the lymph nodes surrounding the superior mesenteric vessels and the abdominal aorta on the first imaging examination. After a thorough examination and laparoscopic exploration, he was diagnosed with stage IV small bowel adenocarcinoma. His clinical course demonstrates that small bowel adenocarcinoma is usually asymptomatic in the early stages and may present with systemic anemia or local obstruction in the late stages. But unlike other small bowel adenocarcinoma patients, our patient presented with a typical mesenteric vascular embolism as the first symptom such as abdominal pain, distension and acid reflux. Therefore, we performed surgery (laparoscopic exploration and small bowel adenocarcinoma resection). During the operation, the tumor was found to have invaded the serosa, and a large mass caused an incomplete obstruction. For localized adenocarcinoma of the small intestine, surgical complete resection (R0) and total regional lymph node resection are the only methods to achieve a curative outcome (7). However, there is still no relevant research data to guide surgical techniques (9). Most small bowel adenocarcinomas with distant metastases can't be removed, and even in some patients with only limited visceral metastases, there is little evidence to support resection of metastases (4).

Combined with postoperative histopathologic and immunohistochemical findings, we gave the patient XELOX regimen in combination with PD-1 blockade. Small bowel adenocarcinoma has traditionally been considered to respond poorly to chemotherapy. Although no randomized trial has demonstrated the benefit of systemic chemotherapy in patients who have distant metastases, several retrospective studies have shown a significant increase in overall and disease-free survival (10–14). A number of studies have evaluated the efficacy of CAPEOX in patients with advanced small bowel adenocarcinoma, and the response rate to this regimen is significantly higher than the rate to conventional therapy 5-fluorouracil/adriamycin/mitomycin C. In terms of safety, the most common side effects of XELOX are neutropenia, complete blood count reduction, nausea and vomiting. In contrast, the incidence of severe side effects of 5-fluorouracil/adriamycin/mitomycin C was extremely high (68%) (15). What's more, the level of Ki67 can predict cancer progression as a biological maker of cell proliferation (16). The patients with high expression of Ki67 have better response to chemotherapy compared to patients with low Ki67 expression. Immunotherapy, such as Bevacizumab and Regorafenib, also plays a potential role due to the microsatellite instability and high tumor burden (17). At present, the chemotherapy regimen of small bowel adenocarcinoma mainly refers to colorectal cancer, mainly fluorouracil. Based on these data, Folfox, CAPEOX with or without Bevacizumab as a first-line treatment for advanced small bowel adenocarcinoma (4).

Recent studies have found that most patients with small bowel adenocarcinoma express high levels of PD-1, suggesting that checkpoint inhibitor therapy may play a role. The Mismatch repair gene (MMR gene), which includes MLH-1, PMS-2, MSH-2, and MSH-6, plays an important part in the repair of DNA homologous recombination repair. MMR gene missing causes deficiency (dMMR), which results in microsatellite instability. Small bowel adenocarcinoma has a higher incidence of dMMR than colorectal cancer, making checkpoint inhibitors an important treatment option for some patients (18). Our patient was diagnosed as MSI-H and dMMR small bowel adenocarcinoma and showed significant benefit after continued treatment with PD-1 blockade. The remaining fused lymph nodes were completely relieved by this therapy. A multicenter, Phase II Pembrolizumab has been shown to be effective in the treatment of patients with advanced small bowel adenocarcinoma and has been shown to be effective in the treatment of refractory dMMR colorectal and non-colorectal tumors such as breast cancer, melanoma and lung cancer (19). At present, there is no report of XELOX in combination with immunotherapy for small bowel adenocarcinoma. Our patient could tolerate this combination without specific side effects during the whole treatment.

In general, the prognosis of advanced small bowel adenocarcinoma is poor. In this case, the patient presents with mesenteric vascular embolism as the initial symptom, which provided an extra basis and thinking for clinical diagnosis of small bowel adenocarcinoma. Palliative surgery can help relieve obstruction in cases where radical surgery is not possible. At the same time, systematic chemotherapy combined with anti-PD-1 therapy can control the MSI-H small bowel adenocarcinoma patient' s disease progression and improve disease-free survival or overall survival.

## Data Availability Statement

The original contributions presented in the study are included in the article/supplementary material, further inquiries can be directed to the corresponding author/s.

## Ethics Statement

Written informed consent was obtained from the individual(s) for the publication of any potentially identifiable images or data included in this article.

## Author Contributions

ZD: drafting the manuscript. XX: acquisition of data. ZZ: conception and design. All authors: revision, adaptation, and final approval of the manuscript.

## Funding

The study was sponsored by the National Natural Science Foundation of China (Grant Numbers: 81972206), which supported data collection, analysis, and manuscript writing.

## Conflict of Interest

The authors declare that the research was conducted in the absence of any commercial or financial relationships that could be construed as a potential conflict of interest.

## Publisher's Note

All claims expressed in this article are solely those of the author and do not necessarily represent those of their affiliated organizations, or those of the publisher, the editors and the reviewers. Any product that may be evaluated in this article, or claim that may be made by its manufacturer, is not guaranteed or endorsed by the publisher.

## Abbreviations

CT: Computed tomography
FDG-PET: Fluorodeoxyglucose-positron emission tomography
PD-1: Programmed cell death protein 1
MSI-H: Microsatellite-unstable
dMMR: deficient mismatch repair.

## References

[1] Siegel RL Miller KD Jemal A. Cancer statistics, 2019. CA Cancer J Clin. (2019) 69:7–34. 10.3322/caac.21551 30620402

[2] Aparicio T Zaanan A Svrcek M Laurent-Puig P Carrere N Manfredi S. Small bowel adenocarcinoma: epidemiology, risk factors, diagnosis and treatment. Dig Liver Dis. (2014) 46:97–104. 10.1016/j.dld.2013.04.013 23796552

[3] Legue LM Bernards N Gerritse SL van Oudheusden TR de Hingh IH Creemers GM. Trends in incidence, treatment and survival of small bowel adenocarcinomas between 1999 and 2013: a population-based study in The Netherlands. Acta Oncol. (2016) 55:1183–9. 10.1080/0284186X.2016.1182211 27170100

[4] Benson AB Venook AP Al-Hawary MM Arain MA Chen YJ Ciombor KK. Small bowel adenocarcinoma, version 1.2020, NCCN clinical practice guidelines in oncology. J Natl Compr Canc Netw. (2019) 17:1109–33. 10.6004/jnccn.2019.0043 31487687 PMC10191182

[5] Barsouk A Rawla P Barsouk A Thandra KC. Epidemiology of cancers of the small intestine: trends, risk factors, and prevention. Med Sci (Basel). (2019) 7:46. 10.3390/medsci7030046 30884915 PMC6473503

[6] Bojesen RD Andersson M Riis LB Nielsen OH Jess T. Incidence of, phenotypes of and survival from small bowel cancer in Denmark, 1994-2010: a population-based study. J Gastroenterol. (2016) 51:891–9. 10.1007/s00535-016-1171-7 26847562

[7] Dabaja BS Suki D Pro B Bonnen M Ajani J. Adenocarcinoma of the small bowel: presentation, prognostic factors, and outcome of 217 patients. Cancer. (2004) 101:518–26. 10.1002/cncr.20404 15274064

[8] Haan JC Buffart TE Eijk PP van de Wiel MA van Wieringen WN Howdle PD. Small bowel adenocarcinoma copy number profiles are more closely related to colorectal than to gastric cancers. Ann Oncol. (2012) 23:367–74. 10.1093/annonc/mdr122 21586687

[9] Zhang S Yuan W Zhang J Chen Y Zheng C Ma J. Clinicopathological features, surgical treatments, and survival outcomes of patients with small bowel adenocarcinoma. Medicine (Baltimore). (2017) 96:e7713. 10.1097/MD.0000000000007713 28767610 PMC5626164

[10] Fishman PN Pond GR Moore MJ Oza A Burkes RL Siu LL. Natural history and chemotherapy effectiveness for advanced adenocarcinoma of the small bowel: a retrospective review of 113 cases. Am J Clin Oncol. (2006) 29:225–31. 10.1097/01coc.0000214931.01062.01 16755174

[11] Halfdanarson TR McWilliams RR Donohue JH Quevedo JF A. single-institution experience with 491 cases of small bowel adenocarcinoma. Am J Surg. (2010) 199:797–803. 10.1016/j.amjsurg.2009.05.037 20609724

[12] Czaykowski P Hui D. Chemotherapy in small bowel adenocarcinoma: 10-year experience of the British Columbia Cancer Agency. Clin Oncol (R Coll Radiol). (2007) 19:143–9. 10.1016/j.clon.2006.12.001 17355111

[13] Koo DH Yun SC Hong YS Ryu MH Lee JL Chang HM. Systemic chemotherapy for treatment of advanced small bowel adenocarcinoma with prognostic factor analysis: retrospective study. BMC Cancer. (2011) 11:205. 10.1186/1471-2407-11-205 21619586 PMC3125281

[14] Aydin D Sendur MA Kefeli U Umut Unal O Tastekin D Akyol M. Evaluation of prognostic factors and treatment in advanced small bowel adenocarcinoma: report of a multi-institutional experience of Anatolian Society of Medical Oncology (ASMO). J BUON. (2016) 21:1242–9. 27837629

[15] Xiang XJ Liu YW Zhang L Qiu F Yu F Zhan ZY. A phase II study of modified FOLFOX as first-line chemotherapy in advanced small bowel adenocarcinoma. Anticancer Drugs. (2012) 23:561–6. 10.1097/CAD.0b013e328350dd0d 22481063

[16] Yang C Zhang J Ding M Xu K Li L Mao L. Ki67 targeted strategies for cancer therapy. Clin Transl Oncol. (2018) 20:570–5. 10.1007/s12094-017-1774-3 29058263

[17] Takayoshi K Kusaba H Uenomachi M Mitsugi K Makiyama C Makiyama A. Suggestion of added value by bevacizumab to chemotherapy in patients with unresectable or recurrent small bowel cancer. Cancer Chemother Pharmacol. (2017) 80:333–42. 10.1007/s00280-017-3371-0 28653251

[18] Klose J Lasitschka F Horsch C Strowitzki MJ Bruckner T Volz C. Prognostic relevance of programmed death-ligand 1 expression and microsatellite status in small bowel adenocarcinoma. Scand J Gastroenterol. (2020) 55:321–9. 10.1080/00365521.2020.1734073 32191146

[19] Le DT Uram JN Wang H Bartlett BR Kemberling H Eyring AD. PD-1 blockade in tumors with mismatch-repair deficiency. N Engl J Med. (2015) 372:2509–20. 10.1056/NEJMoa1500596 26028255 PMC4481136

